# Enhancing the uptake of systematic reviews of effects: what is the best format for health care managers and policy-makers? A mixed-methods study

**DOI:** 10.1186/s13012-018-0779-9

**Published:** 2018-06-22

**Authors:** Christine Marquez, Alekhya Mascarenhas Johnson, Sabrina Jassemi, Jamie Park, Julia E. Moore, Caroline Blaine, Gertrude Bourdon, Mark Chignell, Moriah E. Ellen, Jacques Fortin, Ian D. Graham, Anne Hayes, Jemila Hamid, Brenda Hemmelgarn, Michael Hillmer, Bev Holmes, Jayna Holroyd-Leduc, Linda Hubert, Brian Hutton, Monika Kastner, John N. Lavis, Karen Michell, David Moher, Mathieu Ouimet, Laure Perrier, Andrea Proctor, Thomas Noseworthy, Victoria Schuckel, Sharlene Stayberg, Marcello Tonelli, Andrea C. Tricco, Sharon E. Straus

**Affiliations:** 1 Li Ka Shing Knowledge Institute, St. Michael’s Hospital, Toronto, Canada; 2 BMJ Knowledge Centre, London, UK; 3 Centre Hospitalier Universitaire de Québec (CHUQ), Quebec City, Canada; 4 Department of Medicine, Faculty of Medicine, University of Toronto, Toronto, Canada; 5 Ben Gurion University, Beer Sheva, Israel; 6 Institute of Health Policy, Management and Evaluation, University of Toronto, Toronto, Canada; 7 McMaster Health Forum, Department of Health Research Methods, Evidence and Impact, and Department of Political Science, McMaster University, Hamilton, Canada; 8 Agence de la santé et des services sociaux Montérégie, Longueuil, Quebec City Canada; 9 Ottawa Hospital Research Institute, Ottawa, Canada; 10 School of Epidemiology and Public Health, University of Ottawa, Ottawa, Canada; 11 Ontario Ministry of Health and Long-Term Care, Toronto, Canada; 12 Department of Clinical Epidemiology and Biostatistics, McMaster University, Hamilton, Canada; 13 Department of Community Health Sciences, Cumming School of Medicine, University of Calgary, Calgary, Canada; 14 Department of Medicine, Cumming School of Medicine, University of Calgary, Calgary, Canada; 15 Michael Smith Foundation for Health Research, Vancouver, Canada; 16 Simon Fraser University, Burnaby, BC Canada; 17 Alberta Seniors Health Strategic Clinical Network, Alberta Health Services, Calgary, Canada; 18 Centre Hospitalier Universitaire de Sherbrooke (CHUS), Quebec, Canada; 19 Sinai Health System, Toronto, Canada; 20 Laval University, Quebec City, Canada; 21 Ministry of Health, Victoria, Canada; 22 Alberta Health, Edmonton, Canada; 23 Epidemiology Division, Dalla Lana School of Public Health, University of Toronto, Toronto, Canada

**Keywords:** Knowledge translation, Knowledge synthesis, Decision making, Policy makers, Health care managers, Evidence implementation, Systematic reviews, Usability, Integrated knowledge translation

## Abstract

**Background:**

Systematic reviews are infrequently used by health care managers (HCMs) and policy-makers (PMs) in decision-making. HCMs and PMs co-developed and tested novel systematic review of effects formats to increase their use.

**Methods:**

A three-phased approach was used to evaluate the determinants to uptake of systematic reviews of effects and the usability of an innovative and a traditional systematic review of effects format. In phase 1, survey and interviews were conducted with HCMs and PMs in four Canadian provinces to determine perceptions of a traditional systematic review format. In phase 2, systematic review format prototypes were created by HCMs and PMs via Conceptboard©. In phase 3, prototypes underwent usability testing by HCMs and PMs.

**Results:**

Two hundred two participants (80 HCMs, 122 PMs) completed the phase 1 survey. Respondents reported that inadequate format (Mdn = 4; IQR = 4; range = 1–7) and content (Mdn = 4; IQR = 3; range = 1–7) influenced their use of systematic reviews. Most respondents (76%; *n* = 136/180) reported they would be more likely to use systematic reviews if the format was modified. Findings from 11 interviews (5 HCMs, 6 PMs) revealed that participants preferred systematic reviews of effects that were easy to access and read and provided more information on intervention effectiveness and less information on review methodology. The mean System Usability Scale (SUS) score was 55.7 (standard deviation [SD] 17.2) for the traditional format; a SUS score < 68 is below average usability. In phase 2, 14 HCMs and 20 PMs co-created prototypes, one for HCMs and one for PMs. HCMs preferred a traditional information order (i.e., methods, study flow diagram, forest plots) whereas PMs preferred an alternative order (i.e., background and key messages on one page; methods and limitations on another). In phase 3, the prototypes underwent usability testing with 5 HCMs and 7 PMs, 11 out of 12 participants co-created the prototypes (mean SUS score 86 [SD 9.3]).

**Conclusions:**

HCMs and PMs co-created prototypes for systematic review of effects formats based on their needs. The prototypes will be compared to a traditional format in a randomized trial.

**Electronic supplementary material:**

The online version of this article (10.1186/s13012-018-0779-9) contains supplementary material, which is available to authorized users.

## Background

Knowledge has often been classified as either conceptual (i.e., changes in understanding or attitudes), instrumental (i.e., concrete application), or persuasive (i.e., political or persuasive tool) [1]. The use of knowledge (i.e., evidence) can include any or all of these concepts. In health systems, the use of high-quality research is frequently inadequate, leading to variation in the quality of patient care and cost efficiencies [2, 3]. Although research evidence is generated at a rapid rate, it is not readily available to relevant knowledge users, and when it is available, it is suboptimally applied [4]. One of the major contributors to the suboptimal use of evidence is the volume of research evidence produced [5]. The high volume of research highlights the need for systematic reviews to facilitate knowledge management and evidence uptake (i.e., access and application of knowledge). For policy-makers (PMs) and health care managers (HCMs), evidence is just one component of decision-making and various contextual factors also exist that will influence decision-making; however, this highlights the critical role to ensure that evidence is accessible to decision makers and easily understood.

Systematic reviews summarize all empirical (including qualitative and quantitative) evidence that meets pre-specified eligibility criteria to answer a given research question [6] and can be used for concrete knowledge use. Moreover, since systematic reviews consider the totality of evidence on a topic, they should be the foundational element of knowledge translation (KT) activities [7, 8]. However, evidence indicates that knowledge users such as HCMs and PMs infrequently use systematic reviews in decision-making despite being aware of their potential value [9]. Several studies have been conducted examining this phenomenon and have uncovered key determinants affecting systematic review use including the inability to appraise and understand them, difficulty accessing them among other factors. In a scoping review conducted by Tricco et al., extrinsic and intrinsic barriers and facilitators to systematic reviews use were identified [10]. Extrinsic barriers (i.e., barriers external to the use of systematic reviews) included knowledge users’ lack of time to locate and retrieve systematic reviews and/or their lack of skills to evaluate and apply relevant evidence. In contrast, extrinsic facilitators included establishing collaborations between researchers and PMs to create systematic reviews in a timely manner that are relevant to users. Intrinsic barriers (i.e., barriers inherent to the systematic review) described were issues such as reviews being lengthy and poorly presented, content not addressing relevant questions, and findings not contextualized. One-page summaries of review that included specific content relevant to the audience were reported as intrinsic facilitators.

To address the research-to-policy gap, studies on strategies to promote systematic reviews use have accelerated in growth [11–13]. Many of the strategies identified, however, focus on extrinsic factors to systematic uptake while there is limited research on the impact of strategies that target intrinsic factors such as the format of systematic reviews to increase use. The overall goal of our work is to enhance the uptake of systematic reviews of intervention effects. Given that substantial barriers to systematic review uptake are attributed to lengthy reviews that include irrelevant content, findings lacking contextualization, and facilitators such as shorten summaries promote use [14], our objectives were to collaborate with knowledge users (i.e., HCMs and PMs) to identify intrinsic barriers and facilitators to using systematic reviews of effects and to develop and test the usability of prototypes of systematic review of effects formats that better meet the needs of these knowledge users. We defined format as what (i.e., content including population, intervention, comparison, outcomes, setting, methods) and how (e.g., layout) information is presented.

## Methods

### Design/approach

**Fig. 1:**
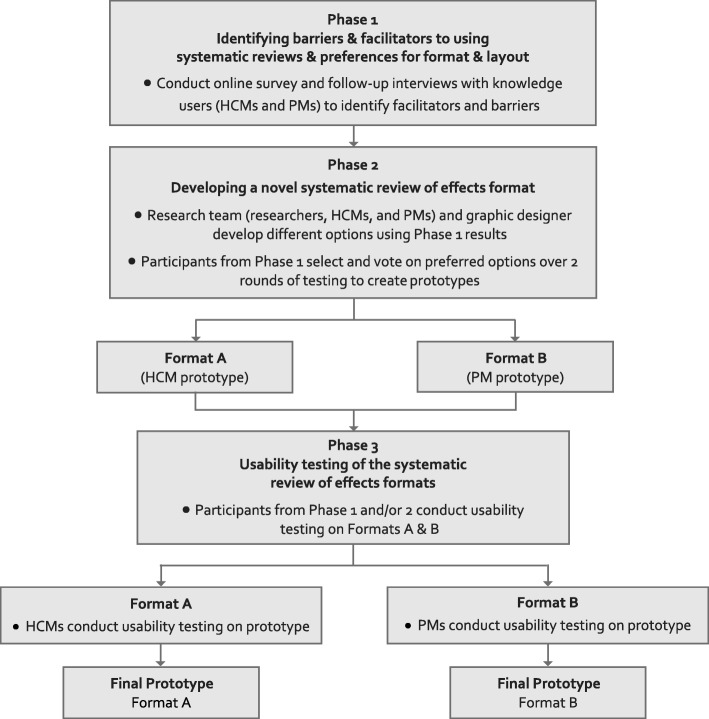
Study flow diagram

A three-phase multiple, mixed-methods approach (Fig. 1) was employed to develop and test prototype formats for optimized presentation of systematic reviews of effects. In phase 1, survey and interviews were conducted with HCMs and PMs to identify the barriers and facilitators to using systematic reviews of effects, preferences for the format and layout of reviews, and the usability of a traditional systematic review of effects format. Phase 2 involved the development of knowledge user informed prototype formats. Phase 3 included interviews with HCMs and PMs to test the usability of the prototype formats.

### Phase 1: identifying key barriers and facilitators to using systematic reviews and preferences for format and layout

Phase 1 involved (i) developing and disseminating the survey; and (ii) conducting interviews with knowledge users.

#### Participants and recruitment

A purposive sampling approach was applied to identify HCMs and PMs from four Canadian provinces (i.e., Alberta, British Columbia, Ontario, and Quebec). These provinces were selected as they account for > 50% of the Canadian population. HCMs were individuals in a managerial or supervisory role in a health care organization with management and supervisory mandates including public health. PMs/advisors/analysts were individuals (non-elected) at some level of government who had some responsibility for analyzing data or making recommendations, and included regional, provincial, and federal representation.

We identified HCMs through hospitals and regional health authorities and PMs through provincial ministry of health agencies.^1^ Potential participants received personalized email invitations to participate in the study from research team members from each of the organizations. To optimize the response rate, a standardized survey method was used [15] and non-respondents were sent email reminders 1, 2, and 3 weeks after initial emails.

#### Development of survey and interview guide

Survey questions (Additional file 1: Appendix S1) were developed from a scoping review of literature on barriers and facilitators (including format and content features) to the use of systematic reviews by HCMs and PMs [10]. Four HCMs and PMs from our project steering committee piloted the questions for face validity. Participants were asked to (a) rank the importance of barriers and facilitators when considering the use of a systematic review in their own setting using a 7-point Likert scale, (b) identify additional barriers and facilitators, (c) indicate preferences for the format and layout of systematic reviews of effects, and (d) indicate if and how often they used systematic reviews in decision-making in the previous year, and whether they received training on interpreting systematic reviews. Survey participants were also asked to indicate whether they would be interested in participating in an interview or future study phases.

Questions in the semi-structured interview guide (Additional file 1: Appendix S2) focused on the usability of traditional systematic reviews (i.e., full-length review selected by the steering committee members) to obtain further information and clarification on participants’ perceptions on clarity, aesthetics, layout, legibility, review structure, and suggestions for improvement. The same interview guide was used for phases 1 and 3; the interview guide was dynamic and refined to include probes on key themes that emerged during interviews. The interview guide also included the System Usability Scale (SUS) questionnaire, a validated scale used to evaluate product usability [16, 17].

#### Data collection and analysis

The survey was administered online to HCMs and PMs in English and French between December 2015 and February 2016 using FluidSurveys™. No identifiers were associated with the data. Analyses of quantitative survey data (e.g., from Likert scale questions) were performed using SPSS 22.0 to calculate descriptive statistics for survey items (e.g., proportions for categorical items, means with standard deviations, and medians with interquartile ranges). We did not test for differences between responses of HCMs and PMs as the survey was intended to describe their use of reviews only. Where appropriate, 7-point Likert scale responses were reduced to the nominal level by combining “1–3” and “5–7” responses into two categories of “accept” and “reject” and keeping “4” as a neutral response. French responses were translated and combined with the English data for analysis. Text responses collected from open-ended survey questions were exported from FluidSurveys™ for qualitative analysis. Applying qualitative content analysis [18], one study team member (SJ) manually grouped responses into categories, while another member of the study team (AMJ) reviewed results for reliability.

Upon survey completion, telephone interviews were conducted with HCMs and PMs from January to February 2016 by two research coordinators (AMJ, SJ); one conducted the interview and the other acted as a note-taker. Using a ‘think aloud’ approach [19], participants were instructed to verbalize all thoughts, actions, and comments while examining a copy of a traditional systematic review of effects [20]. Participants were not asked to apply the document to a relevant health care scenario. Interviews were 30 min and were audio recorded. Both interviewers collectively reviewed the audio files and interview notes. Key findings from each interview were recorded by interviewers to help categorize findings, identify themes, and monitor when thematic saturation (i.e., when little or no new findings are gained from consecutive interviews) was achieved. SUS questionnaire responses were summed and converted to an overall SUS score. SUS scores range from 0 to 100 and a score of > 68 is interpreted as above average usability [16, 17]. Interviews concluded once target numbers were met, and no additional information was gathered.

#### Triangulation

A mixed-methods triangulation technique incorporating quantitative and qualitative methodologies [21–23] was applied using the survey responses and usability testing interviews from phase 1 to uncover convergence, inconsistency, or contradiction. Data collected from both sources were combined and analyzed using a meta-matrix, a secondary analysis technique [24] by two research coordinators (AMJ, SJ). A meta-matrix technique is an approach whereby both quantitative and qualitative data are visual displayed in a matrix framework (i.e., data is displayed along columns and rows) facilitating the combined analysis and data management of quantitative data (i.e., survey) and qualitative data (i.e., interview). It was done once each source was separately analyzed, enabling pattern recognition between the different data types. Intrinsic barriers and facilitators (i.e., those that are inherent to the systematic review) related to format, content, and layout, which emerged from the findings were used to inform the development of a prototype of a systematic review of effects format.

### Phase 2: developing a novel systematic review of effects format

Phase 2 involved (i) developing a prototype and (ii) eliciting feedback from knowledge users.

#### (i) Developing a prototype

Using the phase 1 results, four expert panel members (HCM, PM, and researchers) from the research team and a graphic designer developed different options for the content and format. They provided two content, two layout (e.g., columns), two graphic, and three color combination options. The graphic designer converted these options into 180 building blocks (pieces of content with various graphics and text), which were uploaded onto Conceptboard©, a visual collaboration platform that allows users to anonymously edit content; view and make comments on other users’ content on a real-time ‘whiteboard’, and interact through an anonymous chat feature. This technology has been used to bring together various knowledge users internationally to facilitate work in a collaborative, virtual environment [25].

#### (ii) Eliciting feedback from knowledge users

##### Participants and recruitment

A sequential purposive sampling approach was applied whereby participants from phase 1 who indicated interest in participating in future phases were invited to participate. Participants were provided with several date options and separate into groups based on their availability to participate. Consenting participants were provided with an instructional video on Conceptboard© functionalities and in two rounds, participants reviewed the prototypes, made edits, and commented on participants’ edits.

##### Collaborative-based activity

In round one, participants selected options they preferred from a menu of building blocks and built a new systematic review format. Participants were encouraged to include comments on the rationale behind their selections and to comment on other participants’ boards. In round two, participants submitted votes on the design aspects (i.e., color, layout, content, and favored format) that were tied for preferences in round one. Participants were encouraged to provide comments and discuss their preferences.

#### Data analysis

Two research coordinators (AMJ, SJ) calculated the number of votes (pro, con, conciliatory) by each group (i.e., HCMs and PMs) for each format field, and compared selected options and identified which options were most contentious (i.e. had the most exchanged messages). A quantitative analysis of the number of changes to the revised template format by each group was performed and compared between groups [26–28]. Comments from online discussions were analyzed by one researcher (SJ). Responses were grouped into categories using a content analysis approach [18], and the results were reviewed by a second research coordinator (AMJ). Two different prototype formats: one for HCMs (format A) and one for PMs (format B) were developed based on phase 2 findings.

### Phase 3: usability testing of the systematic review of effects formats

Phase 3 involved conducting interviews with knowledgeable users to evaluate the usability of the novel formats.

#### Participants and recruitment

To test the comprehensiveness and ease of use of the systematic review of effects prototype formats, phase 1 and/or phase 2 participants (i.e., HCMs and PMs) who indicated an interest in being involved in future phases were invited to participate.

#### Data collection and analysis

HCMs and PMs were emailed formats A and B, respectively, on the day of their scheduled usability testing session. Similar to phase 1, a ‘think aloud’ approach [19] was used, and participants were instructed to verbalize all thoughts and actions while examining a copy of the prototype. Sessions were conducted by telephone in November 2016 with one research coordinator facilitating the session and a second acting as a note-taker. All interviews were audio recorded. Audiotapes and notes from each session were analyzed by two research coordinators (AMJ, SJ) using the same method described in phase 1. The SUS scores were determined for formats A and B.

### Ethics and consent

Ethical approval was obtained from St. Michael’s Hospital Research Ethics Board (#15-301) and the Interior Health Authority Research Ethics Board (# 2015-16-68-E). Informed consent was obtained from all participants prior to study activities.

## Results

### Phase 1: identifying key barriers and facilitators to using systematic reviews and preferences for format and layout

#### Participants’ characteristics

A total of 267 survey responses were received, and of these, 202 responses were included in the analyses with 65 excluded due to missing data. Among survey respondents, 80 were identified as HCMs and 122 were identified as PMs. Survey respondents were primarily from Ontario (41%; *n* = 83) and British Columbia (35%; *n* = 71) with 67% (*n* = 136) working in a provincial setting. At least half of survey respondents (55%; *n* = 111) indicated that they received some training on how to read and interpret systematic reviews, and systematic reviews were most often used every 2–3 months (26%; *n* = 53). Characteristics of survey respondents are provided in Table 1.

**Table 1:** Characteristics of study participants

	*n*/%				
	Phase 1		Phase 2		Phase 3 (*n* = 12)
Characteristics^a^	Survey (*n* = 202)	Interview (*n* = 11)	Round 1 (*n* = 13)	Round 2 (*n* = 21)	
Professional role					
Health care manager	80 (39.6)	5 (45.5)	5 (38.5)	9 (42.9)	5 (41.7)
Policy-maker/advisor/analyst	122 (60.4)	6 (54.5)	8 (61.5)	12 (57.1)	7 (58.3)
Provinces					
Alberta	39 (19.3)	3 (27.3)	1 (7.7)	3 (14.3)	3 (25.0)
British Columbia	71 (35.1)	2 (18.2)	5 (38.5)	8 (38.1)	3 (25.0)
Ontario	83 (41.1)	6 (54.5)	7 (53.8)	9 (42.9)	6 (50.0)
Quebec	9 (4.4)	0 (0.0)	0 (0.0)	1 (4.8)	0 (0.0)
Years of experience in role					
1	12 (5.9)	0 (0.0)	1 (7.7)	2 (9.5)	2 (16.7)
2–10	88 (43.6)	7 (63.6)	9 (69.2)	9 (42.9)	2 (16.7)
11–20	59 (29.2)	4 (36.4)	3 (23.1)	8 (38.1)	6 (50.0)
20+	43 (21.3)	0 (0.0)	0 (0.0)	2 (9.5)	2 (16.7)
Level of organization					
National	8 (4.0)	1 (9.1)	2 (15.4)	2 (9.5)	1 (8.3)
Provincial	136 (67.3)	9 (81.8)	10 (76.9)	15 (71.4)	8 (66.7)
Regional	34 (16.8)	0 (0.0)	0 (0.0)	1 (4.8)	2 (16.7)
Local	24 (11.9)	1 (9.1)	1 (7.7)	3 (14.3)	1 (8.3)
Description of organization					
Health service provider	92 (45.5)	5 (45.5)	5 (38.5)	10 (47.6)	6 (50.0)
Government agency	73 (36.1)	2 (18.2)	3 (23.1)	6 (28.6)	4 (33.3)
Funding agency	1 (0.5)	0 (0.0)	0 (0.0)	0 (0.0)	0 (0.0)
Not for profit	3 (1.5)	0 (0.0)	0 (0.0)	0 (0.0)	0 (0.0)
Other	33 (16.3)	4 (36.4)	5 (38.5)	5 (23.8)	2 (16.7)
Geographic location					
Urban	176 (87.1)	11 (100.0)	13 (100.0)	20 (95.2)	12 (100.0)
Suburban	13 (6.4)	0 (0.0)	0 (0.0)	1 (4.8)	0 (0.0)
Rural	13 (6.4)	0 (0.0)	0 (0.0)	0 (0.0)	0 (0.0)
Training on interpreting systematic reviews					
Yes	111 (55.0)	7 (63.3)	9 (69.2)	13 (61.9)	8 (66.7)
No	91 (45.9)	4 (36.4)	4 (30.8)	8 (38.1)	4 (33.3)
Use of systematic reviews					
Never	37 (18.3)	1 (9.1)	0 (0.0)	2 (9.5)	0 (0.0)
Once	27 (13.7)	2 (18.2)	2 (15.4)	3 (14.3)	1 (8.3)
Every 2–3 months	53 (26.2)	4 (36.4)	6 (46.2)	6 (28.6)	3 (25.0)
Once a month	33 (16.3)	2 (18.2)	1 (7.7)	2 (9.5)	1 (8.3)
Twice a month	22 (10.9)	1 (9.1)	0 (0.0)	1 (4.8)	2 (16.7)
Once a week	18 (8.9)	0 (0.0)	2 (15.4)	2 (9.5)	1 (8.3)
Every day	12 (5.9)	1 (9.1)	2 (15.4)	5 (23.8)	4 (33.3)

^a^Note: a sequential sampling approach was used to recruit participants throughout the three phases of this study. Therefore, participants may appear in one or more phases of the study

Survey respondents were asked about their familiarity, knowledge, skill, and belief with respect to systematic reviews (Table 2). Most survey respondents reported that they were familiar with systematic reviews (Mdn = 5; IQR = 2; range = 1–7) and believed systematic reviews can have a practical impact on the decisions they make at work (Mdn = 6; IQR = 2; range = 1–7). Overall, survey respondents stated they had a high level of knowledge about the different types of evidence available to support decision-making (Mdn = 5; IQR = 1; range = 1–7) while a smaller proportion of survey respondents reported they had high level of knowledge on how to appraise the quality of systematic reviews (Mdn = 5; IQR = 2; range = 1–7). Of the survey respondents, 11 (5 HCMs and 6 PMs) consented and participated in follow-up interviews. Survey respondents were invited to participate in all phases of the study; therefore, participants may appear in one or more phases. Characteristics of interview participants as well as participants of other phases of the study (i.e., phases 2 and 3) are described in Tables 1 and 2.

**Table 2:** Study participants’ ratings of familiarity, knowledge, skill, and belief with systematic reviews [Mdn^a^ (IQR^b^); range: (min, max)]

	Score (Mdn^a^) (IQR^b^)); range: (min, max)				
	Phase 1		Phase 2		Phase 3
Response	Survey	Interview	Round 1	Round 2	
Familiarity^c^					
How familiar are you with systematic reviews	5 (2); (1, 7)	5 (1.5); (4, 7)	7 (2); (4, 7)	7 (2); (4, 7)	7 (1); (4, 7)
Belief^d^					
I believe that systematic reviews can have a practical impact on the decision I make at work	6 (2); (1, 7)	5 (2); (5, 7)	6 (1); (5, 7)	6 (1); (4, 7)	7 (1); (5, 7)
Knowledge^d^					
I have high level of knowledge about how to appraise the quality of systematic reviews	5 (2); (1, 7)	5 (2); (2, 7)	6 (2); (2, 7)	6 (2); (2, 7)	6 (0); (2, 7)
I have high level of knowledge about the different types of research evidence available to support decision making	5 (1); (1, 7)	5 (1); (3, 7)	6 (2); (3, 7)	6 (2); (3, 7)	6.5 (1); (3, 7)
Skill^d^					
I am confident in my ability to read and appraise the quality of systematic reviews	5 (2); (1, 7)	5 (2); (2, 7)	5 (2); 2 (7)	5 (2); (2, 7)	6 (1); (2, 7)

^a^Mdn = median

^b^IQR = interquartile range

^c^Likert scale range where 1 = not at all familiar and 7 = extremely familiar

^d^Likert scale range where 1 = strongly disagree and 7 = strongly agree

#### Barriers and facilitators affecting systematic review uptake

##### Format and content

To assess barriers and facilitators to uptake of systematic reviews, survey respondents were provided with a list of scenarios and asked to rate the level of impact on a 7-point Likert scale (Table 3). Format and content were considered one of the key intrinsic factors affecting the use of systematic reviews. Survey respondents reported barriers such as difficulties with format and content not meeting their needs which would affect their decision to use systematic reviews. Similarly, in open-ended responses, challenges with interpreting results and their application were reported as factors limiting use. However, in circumstances where review content addressed their needs and participants liked the format, respondents reported these factors would have a major impact and they would be encouraged to use systematic reviews.

**Table 3:** Barriers and facilitators to use of systematic reviews

Response^a^	Score (Mdn^b^ (IQR^c^))
Barriers	
I find the format of systematic reviews makes them difficult to read	4 (4); (1, 7)
The content of the systematic review does not meet my needs	4 (3); (1, 7)
I do not have the resources to implement evidence from systematic reviews at work	4 (3); (1, 7)
I do not have the skills to appraise systematic reviews for their validity	4 (3); (1, 7)
I do not believe that the outcome reported in a systematic review will actually happen	4 (3); (1, 7)
I don’t agree with the results of a specific systematic review	3 (3); (1, 7)
I am not motivated to use the results of a systematic review in my decision making	3 (3); (1, 7)
I am not familiar with systematic reviews	3 (3); (1, 7)
Facilitators	
The content of most systematic reviews meets my needs	6 (3); (1, 7)
I have the resources to implement evidence from systematic reviews at work	6 (3); (1, 7)
I am part of collaborations between researchers & policy makers/health care managers	6 (3); (1, 7)
I am motivated to use the results of a systematic review in my decision making	6 (1); (1, 7)
I like the format of systematic reviews	5 (2); (1, 7)
I do have the skills to appraise systematic reviews for their validity	5 (2); (1, 7)
I believe that the outcome reported in a systematic review will actually happen	5 (2); (1, 7)
I am familiar with systematic reviews	5 (1.5); (1, 7)
I agree with the results of a specific systematic review	4 (4); (1, 7)

^a^Survey respondents were asked to indicate the level of impact on a Likert scale where 1 = no impact and 7 = major impact

^b^Mdn = median

^c^IQR = interquartile range

##### Resources

Resources were a prominent extrinsic factor perceived to impact use of systematic reviews. Respondents reported lack of resources such as time to search/analysis systematic reviews, availability of relevant systematic review, and organizational support to implement evidence (i.e., leadership not valuing aspects of systematic reviews) which would hinder the use of systematic reviews. In contrast, having adequate resources would facilitate uptake.

##### Motivation, knowledge, and skills

Of the listed potential factors influencing systematic review use, personal motivation, familiarity with systematic reviews, and possessing critical appraisal skills were also viewed as key extrinsic enablers to systematic reviews use.

### Content preference of systematic reviews

Survey respondents rated their level of interest in each of the common content elements in a traditional systematic review. Over 80% of survey respondents rated the conclusion, discussion, strengths/limitations, and results as the review content of most interest. Elements of less interest (i.e., < 50%) were acknowledgements, forest plot diagrams, and conflicts of interest (Additional file 2: Figure SA1).

Follow-up interviews with a portion of survey respondents (i.e., HCMs and PMs) aligned with survey findings. In addition, interview findings suggested possible differences in format and content preferences between HCMs and PMs. In reviewing the traditional systematic review of effects [20], both HCMs and PMs expressed that there was too much information, some repetition, and not enough lay language. HCMs described that they often sought evidence for a specific purpose and that evidence had to be timely, easy to access, and filtered (Table 4). Similarly, PMs cited that that they passed on information from systematic reviews of effects to their superiors in very small, concise amounts and the current format did not suit their needs.

**Table 4:** Content preference of systematic reviews

“Often it’s the ministry that wants an answer or yeah, there is a very vocal patient population or an outbreak or something and we need to understand the evidence immediately”–HCM interview participant	

Majority of participants considered the results and interpretation as the most interesting part of the systematic review of effects. In the results, participants reported that they preferred a high-level summary and were most interested in what interventions were found to be effective and which populations responded favorably to the interventions. They were not interested in interventions that showed no significant effect or in any findings pertaining to search results and study characteristics. Additionally, PM participants stated that they often feel deterred from reading anything with statistical information (e.g., confidence intervals) and only a few liked having forest plot graphs, if they were clear and easy to understand. In the interpretation, most participants found the language in the traditional systematic review of effects to be clear and succinct; however, they wanted more information on next steps/recommendations and possible implementation. PM participants preferred more information on intervention cost included and were concerned with information such as the implementation of the intervention that would assist in decision-making (e.g., the dose of intervention and the effects and duration of the intervention). Sections of information participants were least interested in or felt that is was less of a priority included the limitations and findings related to systematic review methods and the risk of bias. The cited information pertaining to risk of bias could be excluded, as the details could be difficult for some knowledge users to interpret and that the methods section was not as relevant, and they were not interested in reading information on how searches were performed or how articles were screened (Table 5). Moreover, HCM participants stated that they were not interested in study characteristics.

**Table 5:** Content preference of systematic reviews

“…because it’s published in a reputable journal I know that it’s methodical and it’s met all of the criteria, but I don’t need to know all of the details”–PM interview participant	

### Content modifications to systematic reviews

Over 50% of survey respondents rated all suggested review modifications as important (providing ratings from 5 to 7 on a scale from 1 to 7) (Additional file 2: Figure SA2). Overall, 87% (*n* = 147) of survey respondents reported that they would be more likely to use systematic reviews in their decision-making if the content was modified. Suggestions for modification included clear indications of outcomes of potential application in health care/policy and practice, ‘take home messages’ in plain language, summary sections on the impact on/relevance to, health care/policy/practice, and greater focus on the interpretations of results.

#### Format preference of systematic reviews

Regarding traditional review formats, of the features that survey participants were satisfied with only four of the nine format features were rated above 55%. Most participants reported being displeased with the total length of the review, number and length of the tables, and size and location of the journal logo. Format features such as the use of tables and columns to display and organize information, illustration of citations and articles screened, and header/footer to display location in a journal were satisfactory (Additional file 2: Figure SA3). Findings from HCM and PM interviews supported the survey data; specifically, participants liked the use of columns, yet they found them confusing to read and difficult to navigate when the section continued across pages, and figures such as forest plots were hard to comprehend (Table 6).

**Table 6:** Format preference of systematic reviews

“there seem to be some academic literature based formatting requirements that are different from the format/kind of requirements from someone on the policy side would want in a document, so we are trying to use something for, it’s a square peg in a round hole type of thing where something that is built for one purpose is trying to be used for another purpose. So from that perspective it doesn’t work very well”–PM interview participant	

#### Format modifications to systematic reviews

In assessing modifications to a traditional systematic review, over 65% of respondents reported that all suggested modifications were important. Most survey respondents (76%; *n* = 136) reported that they would be more likely to use systematic reviews if the format was modified. Over 80% of survey respondents and interviewees agreed that using bullets, graphs and tables to display results, hyperlinks to supporting documents, and summarizing systematic reviews on one page would be instrumental in increasing review uptake (Additional file 2: Figure SA4).

#### Preference for font and color

Overall, participants reported Calibri 11 or 12, and either blue, or black and white as their preferred text character (i.e., font and size) and color (Additional file 2: Table SA1). More than half of respondents preferred to read materials on the computer (56%, *n =* 96) than in print.

#### Usability of traditional format

HCMs found the traditional systematic review of effects format difficult to use and the various tables, boxes, and graphics lacked a sense of cohesiveness. PMs neither disagreed nor agreed on these two statements but did not feel they would utilize the traditional systematic format of effects frequently. The overall mean SUS score of 55.7 (SD 17.2) was below average (Table 7).

**Table 7:** System usability scale (SUS) scores of the traditional systematic review format

	*Mean* ^*a*^ *(SD)*	
Statement	Traditional Format	
	HCMs (*n* = 5)	PMs (*n* = 6)
I think that I would like to use this document frequently	3.6 (0.6)	2.8 (1.2)
I found this document unnecessarily complex	3.4 (1.3)	3.2 (1.0)
I thought this document was easy to use	2.6 (1.3)	3.0 (1.1)
I think that I would need the support of a technical person to be able to use this document	2.0 (0.7)	2.2 (1.6)
I found the various functions of this document (ex: the tables, boxes, graphics, etc.) were very well integrated	2.6 (0.9)	3.0 (1.6)
I thought there was too much inconsistency in the format of this document	2.2 (0.5)	3.3 (0.5)
I would imagine that most people would learn to use this document very quickly	2.4 (0.9)	2.7 (1.2)
I found this document very cumbersome to use	3.0 (1.0)	3.0 (1.1)
I felt very confident using this document	4.0 (1.2)	3.5 (1.2)
I need to learn a lot of things before I could get going with this document	2.4 (1.1)	2.0 (1.1)
Calculated Score	55.5 (16.5)	55.8 (20.8)

^a^Scoring based on a scale from 1 = strongly disagree to 5 = strongly agree. At least 5 items were reversed coded in order to calculate the mean

### Phase 2: developing a novel systematic review format

Over two rounds, participants developed and selected their preferred novel systematic review of effects formats. Participant characteristics are provided in Tables 1 and 2. In round one, 13 participants were separated into three groups based on availability to participate, and over the course of 1 week, built customized systematic reviews of effects. The first group consisted of 3 HCMs and 3 PMs, the second group consisted of 2 HCMs and 1 PM, and the last group consisted of 4 PMs.

Overall, participants displayed various preferences for color, content, and layout with little consensus (Additional file 2: Table SA2). For example, some preferred to have a traditional content order (e.g., title, background, methods), some preferred a novel order with key messages and results on the first page and background information on the second page, and some preferred a mix between these two. There were mixed preferences on detail preferred for each section of the systematic review, color, and the use of columns. Subsequently, three systematic review options were created for round two instead of just one (in consultation with a graphic designer and a usability expert) (Additional file 1: Appendix S3). *Option one* was composed of black and white headers/icons, presented in two-columns, with content reflecting the traditional order of a systematic review, *option two* included blue headers/icons, presented in one-column, with similar content to option one, presented in a new order (e.g., key messages were on the first page, methods on the second page), and *option three* had blue and green headers/icons, presented in one- and two-columns, with emphasis on different content and presented in a new order.

In round two, 21 participants were separated into three groups based on availability (similar to round one) and voted on the design aspects (e.g., color, content, and layout) over 1 week. Group one consisted of 3 HCMs and 7 PMs, group two consisted of 5 HCMs and 4 PMs, and group three consisted of 1 HCM and 1 PM. HCMs and PMs had unique preferences (Additional file 2: Table SA3), and thus, two different formats were created; one targeted to HCMs (format A), and one targeted to PMs (format B) (Additional file 1: Appendix S4). For example, HCMs preferred use of the blue color scheme as it did not distract the reader and provided the option to print in grayscale, while PMs preferred the use of color to highlight different boxes of text. HCMs favored a traditional content order (i.e., option 1) and PMs indicated that the forest plot was not necessary, and the inclusion of methods was not a priority.

### Phase 3: usability testing of the systematic review formats

Interviews were conducted with 5 HCMs and 7 PMs to assess usability (i.e., content, layout, and graphics) of the prototype formats. Each participant was provided with the prototype specifically design to their reported needs (format A [for HCMs] and format B [for PMs]). Of the total participants that participated in usability testing, 92% of participants (i.e., 11 of 12) were involved in phase 2, co-creating the novel formats. Characteristics of interview participants are provided in Tables 1 and 2.

#### Content of the new format (A and B)

HCMs and PMs found the review language was clear and concise. They valued the inclusion of sections such as background, key messages, but exhibited differing opinions on other sections. For instance, while HCMs appreciated the level of detail in the methods and the inclusion of the funding and limitations sections, PMs did not perceive these components as critical information. Suggested improvements to format A expressed by HCMs included the addition of definitions for ‘relative risk (RR)’ and ‘confidence interval (CI)’ in the forest plot, and a link to the data source. For format B, PMs recommended that the publication year and list of authors be added alongside the title, more headings be inserted in the ‘key message’ to separate ideas, and a description of who produced the summary be added.

#### Layout of the new format (A and B)

Most participants (HCMs and PMs) favored the use of bullets throughout. HCMs and PMs liked having information summarized on 1–2 pages while allowing for sufficient ‘white space.’ Furthermore, most HCMs and PMs stated that they preferred to see the information displayed in two columns instead of single column/row.

#### Aesthetics of the new format (A and B)

Most HCMs and PMs provided positive feedback regarding the overall aesthetics of the new formats, stating that they liked the font and colors and the inclusion of icons to illustrate section content. Minor suggestions were provided on ways to further improve the format. For example, both HCMs and PMs cited that to improve accessibility, the use of a lighter background shade against white text for the key message section would be more suitable.

#### Usability of new format

Participants found the novel formats easy to use and felt confident in using the document. The SUS score for both formats A and B was above 80 (Table 8).

**Table 8:** System usability scale (SUS) scores of the prototype formats (A and B)

	Mean^a^ (SD)	
Statement	Format A	Format B
	HCMs^b^ (*n* = 5)	PMs^b^ (*n* = 7)
I think that I would like to use this document frequently	4.6 (0.5)	4.2 (0.5)
I found this document unnecessarily complex	1.3 (0.5)	1.0 (0)
I thought this document was easy to use	4.3 (1.1)	4.4 (0.6)
I think that I would need the support of a technical person to be able to use this document	1.1 (0.4)	1.6 (0.9)
I found the various functions of this document (e.g., the tables, boxes, graphics) were very well integrated	4.0 (0.8)	3.6 (0.9)
I thought there was too much inconsistency in the format of this document	1.9 (1.1)	1.6 (0.6)
I would imagine that most people would learn to use this document very quickly	4.4 (0.5)	4.0 (1.7)
I found this document very cumbersome to use	1.4 (0.5)	1.0 (0)
I felt very confident using this document	4.6 (0.5)	4.4 (0.6)
I need to learn a lot of things before I could get going with this document	1.6 (0.5)	1.2 (0.5)
Calculated Score	85.5 (8.0)	86.4 (11.5)

^a^Scoring based on a scale from 1 = strongly disagree to 5 = strongly agree. At least 5 items were reversed coded in order to calculate the mean

^b^Participants were involved in co-creating formats A and B (i.e., phase 2)

## Discussion

This is the first study to our knowledge that engages HCMs and PMs working in various settings in co-creating novel formats for the presentation of systematic reviews. In examining a specific set of perceived intrinsic barriers and facilitators to the uptake of systematic reviews [10] (i.e., excluding factors identified at the organization structure and system level), we discovered opportunities for modifications to the traditional format that may increase the use of systematic reviews of effects in decision-making for both HCMs and PMs. In phase 1, we found that inadequate content and formatting, lack of time, and difficulties with interpreting and applying results discouraged HCMs and PMs from using systematic reviews of effects. If systematic reviews of effects were summarized in 1–2 pages, using lay language, with key relevant information to guide decision-making, HCMs and PMs reported they were more likely to use them. For example, HCMs and PMs highlighted that there were less interested in seeing information with respect to methods, conflict of interest, and risk of bias included in the prototype formats. However, one should not conclude that knowledge users find these components as unnecessary, but rather they are confident in the rigor and quality of the information and are less interested in seeing the exact details included in systematic review formats. It is important to note that participants did not initiate the systematic review of effects [20], and therefore, this may have influenced their perceptions of the review.

Findings from our study are similar to work by others [29–32] who reported that HCMs and PMs preferred different formats for the presentation of information in order to rapidly scan for relevance in decision-making. Similarly, Vogel and colleagues [33] found in a survey of health system decision-makers that they preferred concise evidence summaries, although many of them had research experience. A recent systematic review on the effectiveness of evidence summaries (*n* = 6 studies) indicated that they are likely easier to understand than complete systematic reviews, but their ability to increase use of reviews is unclear and no difference was noted in effect on knowledge, understanding, or usability [9]. However, our results extended this by examining the needs and preferences across user groups and found different preference for HCMs and PMs. Specifically, during phase 2, HCMs and PMs worked together, and using an online visual platform, participants were tasked with creating a prototype format that would address their needs and potentially overcome some of the intrinsic barriers previously identified. Among the advantages to utilizing the online visual platform (e.g., edit/results displayed in real-time), selections were less likely to be subjective in nature as participants were encouraged to provide justification for their selection and view/comment on others as well. The outcome of the exercise, however, resulted in very little agreement as to the optimal format. It is possible that since HCMs and PMs were separated into smaller groups to participate in the activity (due to their availability), this may have contributed to the lack of consensus. Differences between HCMs and PMs preferences should be explored in future studies. In round two, outcomes from the voting exercise on design features among the three prototype options indicated that HCMs favored the traditional order of content within systematic reviews of effects, whereas PMs favored a novel format. Hence, based on these preferences, two final prototypes were created, format A (for HCMs) and format B (for PMs). Differences in preference between each knowledge user group may be attributed to several factors including differences in level of skill and expertise in interpreting and using evidence and differences in each group’s need for evidence. Feedback in phase 3 on the usability of each of the final prototypes by each of the specific end-user groups proved to be positive. Overall, each group was satisfied and suggested only minor changes to the new formats to further improve uptake. For example, although HCMs and PMs both agreed on the benefits of bullets to illustrate information, each group identified different sections of information as critical and relevant suggesting alternative placements. Our results from the interviews also demonstrated that participants perceived the presence of icons as beneficial. Most participants approved the use of the ‘thumbs up/down’ icon to highlight key messages. However, the use of these particular icons may be interpreted as recommendations by other knowledge users (i.e., those not involved in creating the prototypes), and therefore, we will explore the usability and interpretation of the ‘thumbs up/down’ icons in future trials of the prototypes. Findings from the SUS scores from format A and B also indicated participants perceived each of the prototypes to be above average with respect to ease of use. Taken together, these findings suggest that systematic review content should be customized to meet the needs of different audiences.

### Limitations and strengths

There are a few limitations to this study approach that should be considered. First, our study focuses on systematic reviews of effects excluding systematic reviews of other areas of study (e.g., realist reviews or reviews integrating qualitative and quantitative evidence). However, we believe that our findings may be transferrable to other types of systematic reviews given that the concepts identified and addressed in this study were not specific to the topic of the review. Second, in our sampling strategy, we did not consider variables such as sex/gender, race/ethnicity, and characteristics such as whether individuals were involved in clinical, public health, or health system policies. Therefore, it is possible that our findings may not reflect the diversity of HCMs and PMs; this was not the focus of our study and could be explored in future work. Moreover, we recruited from the four largest Canadian provinces (i.e., Alberta, British Columbia, Ontario, and Quebec) which included a large and diverse group of HCMs and PMs, including those who speak English or French. Third, we used a sequential sampling approach to recruit participants throughout the three phases of this study, which may have impacted the representativeness of our target population and study generalizability. Furthermore, given that 11 of the 12 participants that rated the usability of the prototype formats also were involved in co-creating the format, it is possible that the usability of the formats is overestimated. Fourth, due to the voluntary nature of this study and our sampling strategy, we were unable to determine survey response rate. Thus, it is possible that this study might not reflect views of all possible participants; non-respondents could have had different characteristics and opinions from respondents. However, saturation of themes was reached in interviews. Fifth, the audio recordings collected from phases 1 and 3 interviews were not transcribed due to limited resources nor did the interviewers independently analyze the data. Although transcriptions would have been beneficial in facilitating data management and analysis, field notes and audio files were used and are considered effective in the absence of transcriptions [34]. Moreover, both the interviewer and note taker reviewed both data sources to ensure common interpretation of data and emergent themes. Sixth, phase 3 included 5 HCMs and 7 PMs with interviews conducted in English only, which may not be representative of this population. However, 5 to 8 participants are sufficient to identify up to 85% of critical usability issues [35]. Seventh, although multiple recruitment strategies were used to reach our target population, we experienced difficulties in recruiting health care managers and policy-makers to our study. Eighth, in both phases 2 and 3, the differences that we identified were based on small sample sizes; however, this is indicative of qualitative work. Ninth, while it is the appropriate approach to include the risk of bias when reporting full results of a systematic review, participants opted to omit this level of detail in the prototype formats. Participants expressed difficulties understanding information related to risk of bias and felt that is was not a priority. However, the risk of bias could be reflected in the interpretation as information about the uncertainty in the results was included in the prototypes. Finally, we acknowledge that there are many factors that influence the uptake of systemic reviews [10]; however, we focused only on intrinsic factors (i.e., format which includes what and how content is presented) to review use and we excluded extrinsic factors such as health system context. As mentioned above, preliminary evidence [9, 33] suggests that improvements to format may help to address some of the challenges and facilitate review use and very little effort has focused on optimizing it. Therefore, for feasibility, we concentrated our efforts on intrinsic factors as opposed to including both intrinsic and extrinsic factors.

A key strength of our study was the use of an integrated KT approach [36]. Adopting such an approach increases the likelihood that knowledge users will find the research relevant and useful. In our study, HCMs and PMs were engaged in the conception of the research questions, protocol development, key message development, and the dissemination of results. Another strength to our study was the use of a collaborative online platform (Conceptboard©). This unique tool allowed us to engage participants from four provinces, share ideas, and co-create novel formats to meet their needs.

## Conclusion

To support health care and managerial and policy decision-making, two prototype formats for the presentation of research evidence were co-developed by HCMs and PMs to improve uptake of systematic reviews. Our team will test the formats prospectively in a randomized trial to assess impact of the novel formats on the use of systematic reviews in health care management and policy decision-making.

## Additional files

Additional file 1: **Appendix S1.** Survey questions. **Appendix S2**. Phases 1 and 3 usability testing interview guide. **Appendix S3**. The three options created for round two; and **Appendix S4**. The two final prototypes created from round two). (PDF 4111 kb)

Additional file 2: **Figure SA1**: Phase 1. Content components that were considered of interest; **Figure SA2**: Phase 1. Content component modifications that were considered important; **Figure SA3**: Phase 1. Format features that were considered satisfactory; **Figure SA4**: Phase 1. Format feature modifications that were considered important; **Table SA1**: Phase 1. Preference towards font and color; **Table SA2**: Phase 2. Preference towards color, content, and layout; and **Table SA3**: Phase 2. Voting results towards color, content, and layout. (DOCX 81 kb)

## Abbreviations

AQESSS: Association Québecoise d’éstablissements de Santé and de Services Sociaux
CAHO: Council of Academic Hospitals of Ontario
CI: Confidence interval
HCM: Health care manager
KT: Knowledge translation
Mdn: Median
PM: Policy-maker
QI: Quality improvement
RHA: Regional Health Authorities
RR: Relative risk
SCNs: Strategic Clinical Networks
SD: Standard deviation
SUS: Systems Usability Scale

## Footnotes

1 HCMs were identified through the Strategic Clinical Networks (SCNs) in Alberta, Council of Academic Hospitals of Ontario (CAHO) in Ontario, and Regional Health Authorities (RHA) and Association Québecoise d’éstablissements de Santé and de Services Sociaux (AQESSS) in Quebec, and the regional health authorities via Michael Smith Foundation for Health Research in British Columbia. PMs were identified through Alberta Health, the Ontario Ministry of Health and Long-Term Care, the Quebec Ministry of Health and Social Services, and the British Columbia Ministry of Health
