# Supplementary material for: Enhancing the uptake of systematic reviews of effects: what is the best format for health care managers and policy-makers? A mixed-methods study
Source: Implement Sci. 2018 Jun 22;13:84. doi: 10.1186/s13012-018-0779-9 (PMC6014014; doi:10.1186/s13012-018-0779-9)
Supplement: Supplementary file 1 — Appendix S1. Survey questions. Appendix S2. Phases 1 and 3 usability testing interview guide. Appendix S3. The three options created for round two; and Appendix S4. The two final prototypes created from round two). (PDF 4111 kb) [file 13012_2018_779_MOESM1_ESM.pdf]

**Additional file 1: (Appendix 1 to 4)**

## Appendix S1. Survey Questions

Participant Eligibility

Do you have at least 6 months experience working as either a health care manager or a policy maker?

Please think of your total experience (including previous positions you have held).

- ○ Yes
- ○ No

Part 1: Participant Profile

1. Which of the following best describes you?

- ○ Health Care Manager
- ○ Policy Maker

Part 1: Participant Profile - Health Care Manager

Please answer the following questions while thinking of your primary place of employment.

2. What province do you primarily work in?

- ○ Ontario
- ○ Alberta
- ○ Quebec
- ○ British Columbia

3. Please enter the approximate number of years you have worked as a health care manager.

Please round up to whole numbers.

[ ]

4. What level of organization do you work in?

○ National

○ Provincial

○ Regional

○ Local

5. How would you describe the geographic location of your workplace?

○ Urban

○ Suburban

○ Rural

6. Which of the following best describes the organization you work for?

○ Health service provider

○ Government agency

○ Funding agency

○ Not for profit

○ Other, please specify... ________________________

7. Which of the following best describes the health sector you work in? (select all that apply)

☐ Public

☐ Private

☐ Home care

☐ Community mental health

☐ Emergency & ambulatory care

☐ Continuing care/Extended care

☐ Acute and inpatient care

☐ Rehabilitation

- ☐ Residential care
- ☐ Paramedic services
- ☐ Other, please specify... ______________________

Part 1: Participant Profile - Policy Maker

2. What province do you primarily work in?

- ○ Ontario
- ○ Alberta
- ○ Quebec
- ○ British Columbia

3. Please enter the approximate number of years you have worked as a policy maker.

[ ]

4. What level of organization do you work in?

- ○ National
- ○ Provincial
- ○ Regional
- ○ Local

5. Which of the following best describes the organization you work for?

- ○ Children & youth services
- ○ Community & social services
- ○ Community, safety & correctional services
- ○ Education
- ○ Health & long term care
- ○ Research & innovation

○ Aboriginal affairs

○ Other, please specify... ______________________

6. Which of the following best describes the position you hold within your organization?

○ Entry level

○ Experienced

○ Management

○ Executive

7. Which of the following best describes the type of work you do?

○ Advisor

○ Analyst

○ Manager

○ Director

○ Other, please specify... ______________________

Part 2: Current knowledge of & use of systematic reviews

A systematic review is a comprehensive summary of all the current research studies that are relevant to a specific research question. It is different from traditional research summaries because it has: clear objectives &an explicit rationale for which studies are included in the summary (providing transparency)systematic searching methods to ensure that the selection of studies is not biased

8. How familiar are you with systematic reviews?

| 1 Not at all familiar | 2 | 3 | 4 Somewhat familiar | 5 | 6 | 7 Extremely familiar |
|---|---|---|---|---|---|---|
| ○ | ○ | ○ | ○ | ○ | ○ | ○ |

9. Please select your level of agreement with the following statements.

| | 1 Strongly disagree | 2 | 3 | 4Neither agree nor disagree | 5 | 6 | 7 Strongly agree |
|---|---|---|---|---|---|---|---|
| I have a high level of knowledge about the different types of research | ○ | ○ | ○ | ○ | ○ | ○ | ○ |

| | | | | | | | |
|---|---|---|---|---|---|---|---|
| evidence available to support decision making | | | | | | | |
| I have a high level of knowledge about how to appraise the quality of systematic reviews | ○ | ○ | ○ | ○ | ○ | ○ | ○ |
| I am confident in my ability to read and appraise the quality of systematic reviews | ○ | ○ | ○ | ○ | ○ | ○ | ○ |
| I believe that systematic reviews can have a practical impact on the decisions I make at work. | ○ | ○ | ○ | ○ | ○ | ○ | ○ |

10. Have you received any training on how to read & interpret systematic reviews?

○ Yes

○ No

11. In the last 6 months, how often have you used research evidence from systematic reviews to inform your work?

Please choose the best response from the following drop down menu.

○ Never

○ Once

○ Every 2-3 months

○ Once a month

○ Twice a month

○ Once a week

○ Every day

Part 3: Preferences for systematic review formats

12. CLICK HERE for an example of the current format of systematic reviews. Please rate your level of satisfaction with the following format features of this systematic review.

| | 1 Completely dissatisfied | 2 | 3 | 4 Neither satisfied or dissatisfied | 5 | 6 | 7 Completely satisfied |
|---|---|---|---|---|---|---|---|
| Total length of document (i.e. number of pages) | ○ | ○ | ○ | ○ | ○ | ○ | ○ |
| Use of columns to organize text | ○ | ○ | ○ | ○ | ○ | ○ | ○ |
| Illustration of citations and articles screened (i.e. study flow diagram) | ○ | ○ | ○ | ○ | ○ | ○ | ○ |
| Header/footer displaying article location in journal | ○ | ○ | ○ | ○ | ○ | ○ | ○ |
| Use of tables to display information | ○ | ○ | ○ | ○ | ○ | ○ | ○ |
| Length of each table in document | ○ | ○ | ○ | ○ | ○ | ○ | ○ |
| Number of tables in document | ○ | ○ | ○ | ○ | ○ | ○ | ○ |
| Size of journal logo in top right hand corner | ○ | ○ | ○ | ○ | ○ | ○ | ○ |
| Location of journal logo in top right hand corner | ○ | ○ | ○ | ○ | ○ | ○ | ○ |

13. The following is a list of some ideas for how systematic review formats could be modified so they are easier to read and use. Please rate how important each of these suggested changes is to you.

| | 1 Not at all important | 2 | 3 | 4Neutral | 5 | 6 | 7 Extremely important |
|---|---|---|---|---|---|---|---|
| The systematic review is summarized on 1 page | ○ | ○ | ○ | ○ | ○ | ○ | ○ |
| The logo from the organization that sponsored the review is clearly indicated | ○ | ○ | ○ | ○ | ○ | ○ | ○ |

| | | | | | | | |
|---|---|---|---|---|---|---|---|
| There is ample white space used in the systematic review format | ○ | ○ | ○ | ○ | ○ | ○ | ○ |
| Bullet points are used instead of paragraphs | ○ | ○ | ○ | ○ | ○ | ○ | ○ |
| Simple tables are used (less than 1 page) | ○ | ○ | ○ | ○ | ○ | ○ | ○ |
| Graphics are used to display the results | ○ | ○ | ○ | ○ | ○ | ○ | ○ |
| The colour scheme is attractive to the eye | ○ | ○ | ○ | ○ | ○ | ○ | ○ |
| The format is tailored for specific audiences | ○ | ○ | ○ | ○ | ○ | ○ | ○ |

14. Are there any other format features that may assist you in reading a systematic review?

[ ]

15. If the format of systematic reviews was modified using some of the ideas above, how likely is it that you would use them at work?

| 1Extremely unlikely | 2 | 3 | 4Neutral | 5 | 6 | 7Extremely likely |
|---|---|---|---|---|---|---|
| ○ | ○ | ○ | ○ | ○ | ○ | ○ |

Part 3: Preferences for systematic review formats (Ctd.)

16. Which of the following fonts do you prefer to read the most?

Click here to view examples of each font.

- ○ Helvetica
- ○ Times New Roman
- ○ Garamond
- ○ Arial
- ○ Cambria
- ○ Calibri

○ Unsure

○ No preference

17. Which of the following font sizes do you prefer in the body of a document?

(i.e. not for headings)

○ Size 10

○ Size 11

○ Size 12

○ Unsure

○ No preference

18. Which of the following colour schemes might <u>attract</u> you to read a document?

(check all that apply)

☐ Red

☐ Orange

☐ Yellow

☐ Green

☐ Blue

☐ Purple

☐ Black & White

☐ All of the above

☐ Unsure

19. Which of the following colour schemes might <u>deter</u> you from reading a document?

(check all that apply)

☐ Red

☐ Orange

☐ Yellow

☐ Green

☐ Blue

☐ Purple

☐ Black & White

☐ All of the above

☐ Unsure

20. The following are examples of research summaries in different formats & styles. Please click on each example to view it:

FORMAT A FORMAT B FORMAT C

Please select which format you prefer the most.

Please disregard the content, we are interested in which format & layout appeals to you the most.

○ Format A

○ Format B

○ Format C

21. How do you prefer to read research materials?

○ In Print

○ On my computer (Electronic)

Part 4: Content of systematic reviews

22. CLICK HERE for an example of the current content of systematic reviews. If researchers decided to create a modified (shortened) version of the content in systematic reviews, they would need to know which pieces of information are of most interest to their target audience. Please rate your level of interest in each of the following systematic review components.(Please refer to the provided example if you wish)

| | 1 Not at all interesting | 2 | 3 | 4 Moderately interesting | 5 | 6 | 7 Extremely interesting |
|---|---|---|---|---|---|---|---|
| Introduction | ○ | ○ | ○ | ○ | ○ | ○ | ○ |
| Study flow diagram (pg 3 in example) | ○ | ○ | ○ | ○ | ○ | ○ | ○ |

| | | | | | | | |
|---|---|---|---|---|---|---|---|
| Methods | ○ | ○ | ○ | ○ | ○ | ○ | ○ |
| Characteristics of included studies (Table 1 in example) | ○ | ○ | ○ | ○ | ○ | ○ | ○ |
| Methodological quality of included studies (pg 7 & Table 4 in example) | ○ | ○ | ○ | ○ | ○ | ○ | ○ |
| Data from each included study presented in a table (Tables 2-3 in example) | ○ | ○ | ○ | ○ | ○ | ○ | ○ |
| Results in written text | ○ | ○ | ○ | ○ | ○ | ○ | ○ |
| Discussion | ○ | ○ | ○ | ○ | ○ | ○ | ○ |
| Strengths & limitations | ○ | ○ | ○ | ○ | ○ | ○ | ○ |
| Conclusions | ○ | ○ | ○ | ○ | ○ | ○ | ○ |
| References | ○ | ○ | ○ | ○ | ○ | ○ | ○ |
| Acknowledgements | ○ | ○ | ○ | ○ | ○ | ○ | ○ |
| Conflicts of interest | ○ | ○ | ○ | ○ | ○ | ○ | ○ |
| Forest plot diagram (pg 14-15 in example) | ○ | ○ | ○ | ○ | ○ | ○ | ○ |

23. The following is a list of some <u>ideas</u> for how the content of systematic reviews could be modified so they are easier to read and use.Please rate how important each of these suggested changes is to you.

| | 1 Not at all important | 2 | 3 | 4Neutral | 5 | 6 | 7 Extremely important |
|---|---|---|---|---|---|---|---|
| Title of the review is framed as a question | ○ | ○ | ○ | ○ | ○ | ○ | ○ |
| Applications for policy makers are clearly indicated | ○ | ○ | ○ | ○ | ○ | ○ | ○ |
| Applications for health care practice are clearly indicated | ○ | ○ | ○ | ○ | ○ | ○ | ○ |
| Outcomes of potential applications | ○ | ○ | ○ | ○ | ○ | ○ | ○ |

| | | | | | | | |
|---|---|---|---|---|---|---|---|
| in health care or policy are clearly indicated | | | | | | | |
| Large focus on the interpretation of the results | ○ | ○ | ○ | ○ | ○ | ○ | ○ |
| Consistent approaches used to report results (e.g. effect sizes) | ○ | ○ | ○ | ○ | ○ | ○ | ○ |
| Clear "take home messages" in plain language | ○ | ○ | ○ | ○ | ○ | ○ | ○ |
| Publication date of the review is clearly indicated | ○ | ○ | ○ | ○ | ○ | ○ | ○ |
| Summary sections on the relevance to health care/policy | ○ | ○ | ○ | ○ | ○ | ○ | ○ |
| Summary sections on the impact on health care/policy | ○ | ○ | ○ | ○ | ○ | ○ | ○ |

24. Are there any other content features which may assist you in reading a systematic review?

[ ]

25. If the content from systematic reviews was modified using some of the ideas above, how likely is it that you would use them at work?

| 1Extremely unlikely | 2 | 3 | 4Neutral | 5 | 6 | 7Extremely likely |
|---|---|---|---|---|---|---|
| ○ | ○ | ○ | ○ | ○ | ○ | ○ |

Part 5: Barriers influencing systematic review uptake

26. We are interested in knowing what factors would hinder you from using a systematic review at work. Please indicate the <u>level of impact</u> the following scenarios would have <u>on whether you would use evidence from a systematic review at work</u>.

| | 1No impact | 2 | 3 | 4Neutral | 5 | 6 | 7Major impact |
|---|---|---|---|---|---|---|---|
| I don't agree with the results of a specific systematic review | ○ | ○ | ○ | ○ | ○ | ○ | ○ |
| I don't believe that the outcome reported in a systematic review will actually happen | ○ | ○ | ○ | ○ | ○ | ○ | ○ |

| | | | | | | | |
|---|---|---|---|---|---|---|---|
| I am not motivated to use the results of a systematic review in my decision making | ○ | ○ | ○ | ○ | ○ | ○ | ○ |
| I am not familiar with systematic reviews | ○ | ○ | ○ | ○ | ○ | ○ | ○ |
| I do not have the skills to appraise systematic reviews for their validity | ○ | ○ | ○ | ○ | ○ | ○ | ○ |
| I find the format of systematic reviews makes them difficult to read | ○ | ○ | ○ | ○ | ○ | ○ | ○ |
| The content of most systematic review do not meet my needs | ○ | ○ | ○ | ○ | ○ | ○ | ○ |
| I do not have the resources to implement evidence from systematic reviews at work | ○ | ○ | ○ | ○ | ○ | ○ | ○ |

27. Have you experienced any other barriers (not listed above) to the application of systematic reviews at work?

Part 6: Facilitators influencing systematic review uptake

28. We are interested in knowing what factors would encourage you to use a systematic review at work. Please indicate the level of impact the following scenarios would have on whether you would use evidence from a systematic review at work.

| | 1No impact | 2 | 3 | 4Neutral | 5 | 6 | 7Major impact |
|---|---|---|---|---|---|---|---|
| I agree with the results of a specific systematic review | ○ | ○ | ○ | ○ | ○ | ○ | ○ |
| I believe that the outcome reported in a systematic review will actually happen | ○ | ○ | ○ | ○ | ○ | ○ | ○ |
| I am motivated to use the results of a systematic review in my decision making | ○ | ○ | ○ | ○ | ○ | ○ | ○ |
| I am familiar with systematic reviews | ○ | ○ | ○ | ○ | ○ | ○ | ○ |
| I do have the skills to appraise systematic reviews for their validity | ○ | ○ | ○ | ○ | ○ | ○ | ○ |
| I like the format of systematic reviews | ○ | ○ | ○ | ○ | ○ | ○ | ○ |

| | | | | | | | |
|---|---|---|---|---|---|---|---|
| The content of most systematic reviews meets my needs | ○ | ○ | ○ | ○ | ○ | ○ | ○ |
| I have the resources to implement evidence from systematic reviews at work | ○ | ○ | ○ | ○ | ○ | ○ | ○ |
| I am a part of collaborations between researchers & policy makers/health care managers | ○ | ○ | ○ | ○ | ○ | ○ | ○ |

29. Have you experienced any other facilitators (not listed above) to the application of systematic reviews at work?

## Appendix S2. Phase 1 and 3 usability testing interview guide

1. What are your initial thoughts on this document?
2. Do you like this document? Why or why not?
3. How could the document be improved?

Content Questions:

4. Did you find that the information provided in the document was clear and easy to understand?

*If no,*

   a. What doesn't make sense/isn't clear?
   b. Is the language appropriate?
   c. Is there anything in this tool that you were unsure about or had trouble understanding?

*If yes,*

   d. What aspects of the tool make it easy to understand?

5. Is there anything missing from this document?

*If so,*

   a. What information would you like to see included?
   b. Why would this information be helpful?

6. Is there anything in this tool that you would remove or change?

*If so,*

   a. What?
   b. Why would you change this information?

Aesthetics Questions:

7. How did you find the combination of colours that was used for this document?
8. Would you suggest we change any of the colours?
   a. And if so to what colours?

9. How did you find the style of font that was used for this document?
10. Would you suggest we change the font style?
    a. And if so to what font style?

11. How did you find the font size?
12. Would you suggest we change the font size?
    a. And if so to what size?

Purpose of the document Questions:

13. Would this document help you in your practice? Why or why not?
14. Can you think of any clear barriers you may face when using this document in your practice?

Overall Questions:

15. Do you have any other feedback, comments, or concerns regarding any of the materials we have discussed today that you would like to bring up or share?

16. Do you currently use any summary document related to the use of systematic reviews?
    a. If yes, which ones?

Systems Usability Scale:

1. I think that I would like to use this document frequently

Strongly disagree | Strongly agree

| | | | | |
|---|---|---|---|---|
| | | | | |
| 1 | 2 | 3 | 4 | 5 |

2. I found this document unnecessarily complex

Strongly disagree | Strongly agree

| | | | | |
|---|---|---|---|---|
| | | | | |
| 1 | 2 | 3 | 4 | 5 |

3. I thought this document was easy to use

Strongly disagree | Strongly agree

| | | | | |
|---|---|---|---|---|
| | | | | |
| 1 | 2 | 3 | 4 | 5 |

4. I think that I would need the support of a technical person to be able to use this document

Strongly disagree | Strongly agree

| | | | | |
|---|---|---|---|---|
| | | | | |
| 1 | 2 | 3 | 4 | 5 |

5. I found the various functions of this document (ex: the tables, boxes, graphics, etc.) were very well integrated

| Strongly disagree | | | | Strongly agree |
|---|---|---|---|---|
| | | | | |
| 1 | 2 | 3 | 4 | 5 |

6. I thought there was too much inconsistency in the format of this document

| Strongly disagree | | | | Strongly agree |
|---|---|---|---|---|
| | | | | |
| 1 | 2 | 3 | 4 | 5 |

7. I would imagine that most people would learn to use this document very quickly

| Strongly disagree | | | | Strongly agree |
|---|---|---|---|---|
| | | | | |
| 1 | 2 | 3 | 4 | 5 |

8. I found this document very cumbersome to use

| Strongly disagree | | | | Strongly agree |
|---|---|---|---|---|
| | | | | |
| 1 | 2 | 3 | 4 | 5 |

9. I felt very confident using this document

| Strongly disagree | | | | Strongly agree |
|---|---|---|---|---|
| | | | | |
| 1 | 2 | 3 | 4 | 5 |

10. I need to learn a lot of things before I could get going with this document

| Strongly disagree | | | | Strongly agree |
|---|---|---|---|---|
| | | | | |
| 1 | 2 | 3 | 4 | 5 |

# Appendix S3. The three options created for round two

## Option #1

## A systematic review of the effectiveness of quality improvement (QI) strategies for care coordination to reduce health care utilization.


### Authors:

Tricco A, Antony J, Ivers N, Ashoor H, Khan P, Blondal E, Ghassemi M, MacDonald H, Chen M, Ezer L, Straus S.


### Background:

A small group of patients use health services frequently, and account for a large amount of hospital admissions and ED visits. There is a need to understand the impact of QI strategies aimed at reducing the use of these services in this group.

### Methods:

We searched MEDLINE, Embase and the Cochrane Library and found 36 randomised control trials (RCTs) and 14 companion reports (total 7494 patients) assessing QI strategies for the coordination of care. We used the Cochrane Effective Practice and Organisation of Care Risk-of-Bias Tool. A meta-analysis was conducted on data reported from RCTs. The included studies were published between 1987 and 2014 by researchers in North America, Europe, Australia, Israel, and South Africa.

### Key messages for policy and practice:

The results from this systematic review are similar to previous systematic reviews that found the following:

- Patient education and promotion of self-management are effective for improving care
- Case management and multidisciplinary teams are effective for reducing ED visits

Patient education and promotion of self-management may be less resource intensive than case management; QI strategies targeting patients (as opposed to clinicians) might be a more efficient use of resources.

The lack of effectiveness of QI strategies in patients with mental illnesses may have been because in 7 of the 11 studies involving patients with mental illness, those in the comparison group were also involved in similar QI strategies.

Consider strategies such as team changes, case management and promotion of self-management if you are looking for interventions to reduce hospital admissions and ED visits. Different strategies may be needed for patients with mental illness.

### Definition of care coordination:

Strategies that involve organizing patient care activities between all parties (including the patient) involved in a patient's care to facilitate high-quality delivery of health care services. Many QI strategies involve care coordination.

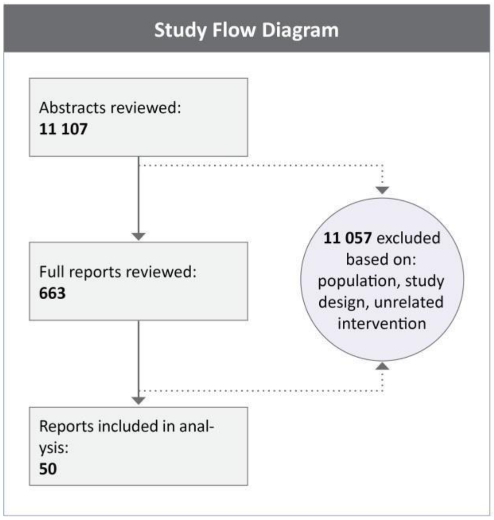


### Results:

Elderly patients who were involved in QI strategies (for a median duration of 9 months) were less likely to visit the ED than elderly patients who were not involved in QI strategies.

Fewer patients with chronic conditions who were involved QI strategies (for a median duration of one year) were less likely to be admitted to hospital than patients with chronic conditions who were not involved in QI strategies.

No change in frequency of hospital admissions was found among patients with mental illness (e.g., schizophrenia and severe bipolar disorder) who were involved in QI strategies.

## The most effective QI strategies were

- Case management:
  Diagnosis, treatment and ongoing patient management (e.g., arranging referrals, follow-up of test results, education, reminders) by an individual other than the primary care clinician.
- Primary care team changes:
  Changes to the team and how it functions, including routine patient visits with personnel other than the primary care physician, use of multidisciplinary staff and the expansion or revision of team members' professional roles
- Promotion of self-management:
  Provision of equipment (e.g., home glucometers) or access to resources (e.g., electronic systems for transferring glucose measurements) and goal-setting to empower patients to manage their own health.
- Patient education:
  Educating patients about their disease, including prevention and treatment.

## QI care coordination strategies that did not have significant effects were:

- Decision support: Provision of feedback to clinicians including supportive evidence or organizational supports to facilitate care coordination.
- Clinical information system: distinguished from administrative information systems by the requirement for data entry or data retrieval by clinicians at the point of care.

QI strategies with a patient navigator component (e.g., a staff person who connects patient with doctors and available therapies) were not found to be effective.

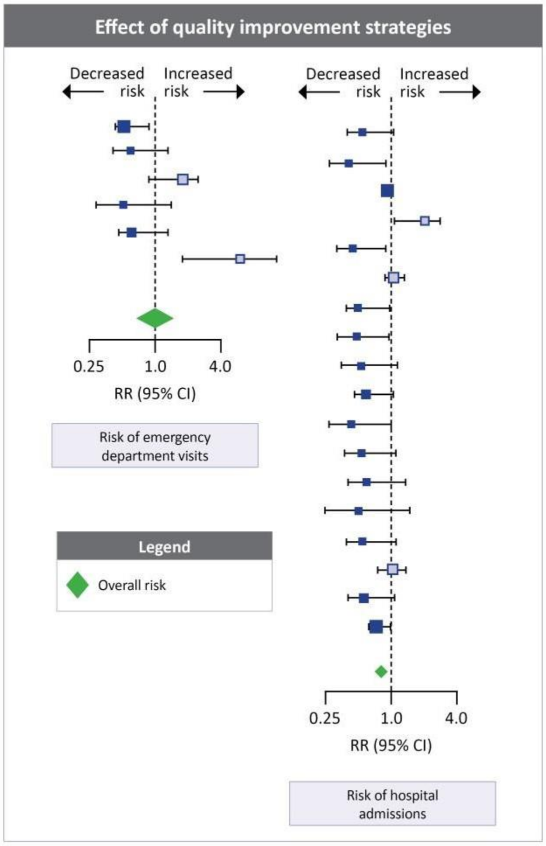


## Limitations:

Studies reported few details about the intensity, dose, and delivery of QI strategies. Our analysis was limited because the QI strategies were complex and difficult to categorize; some of the strategies overlapped over one another, such as case management and team changes. We did not examine data on patient experience and quality of life, because the focus of our research was the health system. We were unable to summarize information on cost in a meaningful manner, because the information varied widely by context.

## Funding:

This project was funded by the Building Bridges to Integrate Care (BRIDGES) initiative, through the Ontario Ministry of Health and Long-Term Care.

## Citation:

Tricco, A. C., Antony, J., Ivers, N. M., Ashoor, H. M., Khan, P. A., Blondal, E., Ghassemi, M., MacDonald, H., Chen, M., Ezer, L., Straus, S. E. (2014). Effectiveness of quality improvement strategies for coordination of care to reduce use of health care services: A systematic review and meta-analysis. Canadian Medical Association Journal, 186(15). doi:10.1503/cmaj.140289

## Recommended reading list:

Click here for the full list of studies included in this systematic review

**Option #2**

# Quality improvement (QI) strategies reduced hospital admissions among adult patients with chronic conditions and reduced emergency department (ED) visits among elderly patients.

## Background:

Frequent users of health services are a small group of patients who account for a large amount of hospital admissions and ED visits. There is a need to understand the impact of QI care coordination strategies aimed at reducing the use of these services in this group.

## Key messages for policy and practice:

The results from this systematic review are similar to previous systematic reviews that found the following:

- Patient education and promotion of self-management are effective for improving care
- Case management and multidisciplinary teams are effective for reducing ED visits

Patient education and promotion of self-management may be less resource intensive than case management; QI strategies targeting patients (as opposed to clinicians) might be a more efficient use of resources.

We observed statistically significant reductions in ED visits among elderly patients, but not specifically for case management or team changes.

The lack of effectiveness of QI strategies in patients with mental illnesses may have been because in 7 of the 11 studies involving patients with mental illness, those in the comparison group were also involved in similar QI strategies.

Consider strategies such as team changes, case management and promotion of self-management if you are looking for interventions to reduce hospital admissions and ED visits from chronic disease patients and elderly patients. Different strategies may be needed for patients with mental illness. It still remains unclear how decision makers can best use care coordination strategies for other patient subgroups and across various settings.

## Results:

Elderly patients who were involved in QI strategies (for a median duration of 9 months) were less likely to visit the ED than elderly patients who were not involved in QI strategies.

Fewer patients with chronic conditions who were involved QI strategies (for a median duration of one year) were less likely to be admitted to hospital than patients with chronic conditions who were not involved in QI strategies.

No change in hospital admissions was found among patients with mental illness (e.g., schizophrenia and severe bipolar disorder) who were involved in QI strategies.

## The most effective QI strategies were:

- **Case management:** Diagnosis, treatment and ongoing patient management (e.g., arranging referrals, follow-up of test results, education, reminders) by an individual other than the primary care clinician.
- **Primary care team changes:** Changes to the team and how it functions, including routine patient visits with personnel other than the primary care physician, use of multidisciplinary staff and the expansion or revision of team members' professional roles
- **Promotion of self-management:** Provision of equipment (e.g., home glucometers) or access to resources (e.g., electronic systems for transferring glucose measurements) and goal-setting to empower patients to manage their own health.
- **Patient education:** Educating patients about their disease, including prevention and treatment.

## The most effective QI strategies had the following:

- An outreach component that included patient assessment, education, and follow-up conducted outside the clinic or hospital
- A target population that included patients who were the most frequent users of the health care system and those who were at risk of becoming the most frequent users

## QI care coordination strategies that did not have significant effects were:

- Decision support: Provision of feedback to clinicians including supportive evidence or organizational supports to facilitate care coordination.
- Clinical information system: distinguished from administrative information systems by the requirement for data entry or data retrieval by clinicians at the point of care.

QI strategies with a patient navigator component (e.g., a staff person who connects patient with doctors and available therapies) were not found to be effective.

## Methods:

We searched MEDLINE, Embase and the Cochrane Library and found 36 randomised control trials (RCTs) and 14 companion reports (total 7494 patients) assessing QI strategies for the coordination of care. We used the Cochrane Effective Practice and Organisation of Care Risk-of-Bias Tool. A meta-analysis was conducted on data reported from RCTs. The included studies were published between 1987 and 2014 by researchers in North America, Europe, Australia, Israel, and South Africa.

## Limitations:

Studies reported few details about the intensity, dose, and delivery of QI strategies.

## Funding:

This project was funded by the Building Bridges to Integrate Care (BRIDGES) initiative, through the Ontario Ministry of Health and Long-Term Care.

## Authors:

Tricco A, Antony J, Ivers N, Ashoor H, Khan P, Blondal E, Ghassemi M, MacDonald H, Chen M, Ezer L, Straus S.

## Citation:

Tricco, A. C., Antony, J., Ivers, N. M., Ashoor, H. M., Khan, P. A., Blondal, E., Ghassemi, M., MacDonald, H., Chen, M., Ezer, L., Straus, S. E. (2014). Effectiveness of quality improvement strategies for coordination of care to reduce use of health care services: A systematic review and meta-analysis. Canadian Medical Association Journal, 186(15). doi:10.1503/cmaj.140289

**Option #3**

## Quality improvement (QI) strategies reduced hospital admissions among adult patients with chronic conditions and reduced emergency department (ED) visits among elderly patients.

### Key messages for policy and practice:

The results from this systematic review are similar to previous systematic reviews that found the following:

- Patient education and promotion of self-management are effective for improving care
- Case management and multidisciplinary teams are effective for reducing ED visits

Patient education and promotion of self-management may be less resource intensive than case management; QI strategies targeting patients (as opposed to clinicians) might be a more efficient use of resources.

The lack of effectiveness of QI strategies in patients with mental illnesses may have been because in 7 of the 11 studies involving patients with mental illness, those in the comparison group were also involved in similar QI strategies.

Consider strategies such as team changes, case management and promotion of self-management if you are looking for interventions to reduce hospital admissions and ED visits. Different strategies may be needed for patients with mental illness.

### The most effective QI strategies were:

- **Case management:** Diagnosis, treatment and ongoing patient management (e.g., arranging referrals, follow-up of test results, education, reminders) by an individual other than the primary care clinician.
- **Primary care team changes:** Changes to the team and how it functions, including routine patient visits with personnel other than the primary care physician, use of multidisciplinary staff and the expansion or revision of team members' professional roles
- **Promotion of self-management:** Provision of equipment (e.g., home glucometers) or access to resources (e.g., electronic systems for transferring glucose measurements) and goal-setting to empower patients to manage their own health.
- **Patient education:** Educating patients about their disease, including prevention and treatment.

### The most effective QI strategies had the following:

- An outreach component that included patient assessment, education, and follow-up conducted outside the clinic or hospital
- A target population that included patients who were the most frequent users of the health care system and those who were at risk of becoming the most frequent users

### QI care coordination strategies that did not have significant effects were:

- Decision support: Provision of feedback to clinicians including supportive evidence or organizational supports to facilitate care coordination.
- Clinical information system: distinguished from administrative information systems by the requirement for data entry or data retrieval by clinicians at the point of care.

QI strategies with a patient navigator component (e.g., a staff person who connects patient with doctors and available therapies) were not found to be effective.

## Background:

A small group of patients use health services frequently, and account for a large amount of hospital admissions and ED visits. There is a need to understand the impact of QI strategies aimed at reducing the use of these services in this group.

## Definition of care coordination:

Strategies that involve organizing patient care activities between all parties (including the patient) involved in a patient's care to facilitate high-quality delivery of health care services. Many QI strategies involve care coordination.

## Results:

Elderly patients who were involved in QI strategies (for a median duration of 9 months) were less likely to visit the ED than elderly patients who were not involved in QI strategies.

Fewer patients with chronic conditions who were involved QI strategies (for a median duration of one year) were less likely to be admitted to hospital than patients with chronic conditions who were not involved in QI strategies.

No change in frequency of hospital admissions was found among patients with mental illness (e.g., schizophrenia and severe bipolar disorder) who were involved in QI strategies.

T able 1.
The effect of quality improvement strategies on emergency department visits, hospital admissions, clinic visits, and length of stay

| Health care event | Relative Risk (95% Confidence Interval) | Number of included studies |
|---|---|---|
| Emergency department visits | **1.11** (0.65 to 1.90) | **6** |
| Hospital admissions | **0.81** (0.72 to 0.91) | **18** |
| Clinic visits | **0.86** (0.58 to 1.27) | **5** |


## Funding:

This project was funded by the Building Bridges to Integrate Care (BRIDGES) initiative, through the Ontario Ministry of Health and Long-Term Care.


## Citation:


Tricco, A. C., Antony, J., Ivers, N. M., Ashoor, H. M., Khan, P. A., Blondal, E., Ghassemi, M., MacDonald, H., Chen, M., Ezer, L., Straus, S. E. (2014). Effectiveness of quality improvement strategies for coordination of care to reduce use of health care services: A systematic review and meta-analysis. Canadian Medical Association Journal, 186(15). doi:10.1503/cmaj.140289


## Authors:


Tricco A, Antony J, Ivers N, Ashoor H, Khan P, Blondal E, Ghassemi M, MacDonald H, Chen M, Ezer L, Straus S.


## Recommended reading list:

Click here for the full list of studies included in this systematic review

## Appendix S4. The two final prototypes created from round two

### Health care manager novel format (Format A)

# Key strategies for care coordination to reduce healthcare utilization: a systematic review

Tricco A, Antony J, Ivers N, Ashoor H, Khan P, Blondal E, Ghassemi M, MacDonald H, Chen M, Ezer L, Straus S.

### Background:

A small group of patients use health services frequently, and account for a large amount of hospital admissions and ED visits. There is a need to understand the impact of QI strategies aimed at reducing the use of these services in this group.

### Definition of care coordination:

Strategies that involve organizing patient care activities between all parties (including the patient) involved in a patient's care to facilitate high-quality delivery of health care services. Many QI strategies involve care coordination.

### Methods:

We searched MEDLINE, Embase and the Cochrane Library and found 36 randomised control trials (RCTs) and 14 companion reports (total 7494 patients) assessing QI strategies for the coordination of care. We used the Cochrane Effective Practice and Organisation of Care Risk-of-Bias Tool. A meta-analysis was conducted on data reported from RCTs. The included studies were published between 1987 and 2014 by researchers in North America, Europe, Australia, Israel, and South Africa.

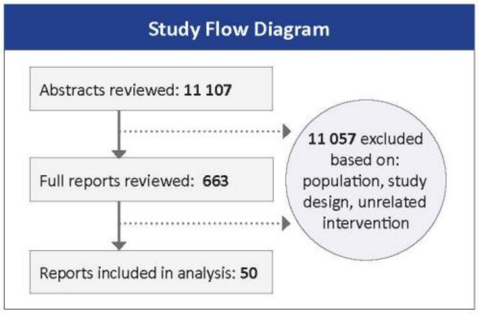


### The most effective QI strategies were:

- Case management:
  Diagnosis, treatment and ongoing patient management (e.g., arranging referrals, follow-up of test results, education, reminders) by an individual other than the primary care clinician.
- Primary care team changes:
  Changes to the team and how it functions, including routine patient visits with personnel other than the primary care physician, use of multidisciplinary staff and the expansion or revision of team members' professional roles
- Promotion of self-management:
  Provision of equipment (e.g., home glucometers) or access to resources (e.g., electronic systems for transferring glucose measurements) and goal-setting to empower patients to manage their own health.
- Patient education:
  Educating patients about their disease, including prevention and treatment.

### QI care coordination strategies that did not have significant effects were:

- Decision support:
  Provision of feedback to clinicians including supportive evidence or organizational supports to facilitate care coordination.
- Clinical information system:
  Distinguished from administrative information systems by the requirement for data entry or data retrieval by clinicians at the point of care.
- QI strategies with a patient navigator component (e.g., a staff person who connects patient with doctors and available therapies) were not found to be effective.

### Key messages for policy and practice:

The results from this systematic review are similar to previous systematic reviews that found the following:

- Patient education and promotion of self-management are effective for improving care
- Case management and multidisciplinary teams are effective for reducing ED visits

Patient education and promotion of self-management may be less resource intensive than case management; QI strategies targeting patients (as opposed to clinicians) might be a more efficient use of resources.

The lack of effectiveness of QI strategies in patients with mental illnesses may have been because in 7 of the 11 studies involving patients with mental illness, those in the comparison group were also involved in similar QI strategies.

Consider strategies such as team changes, case management and promotion of self-management if you are looking for interventions to reduce hospital admissions and ED visits. Different strategies may be needed for patients with mental illness. It still remains unclear how decision makers can best use care coordination strategies for other patient subgroups and across various settings.

## Results:

Elderly patients who were involved in QI strategies (for a median duration of 9 months) were less likely to visit the ED than elderly patients who were not involved in QI strategies.

Fewer patients with chronic conditions who were involved QI strategies (for a median duration of one year) were less likely to be admitted to hospital than patients with chronic conditions who were not involved in QI strategies.

No change in frequency of hospital admissions was found among patients with mental illness (e.g., schizophrenia and severe bipolar disorder) who were involved in QI strategies.

## Quantitative data source:

Click here to view Table 1. The effect of quality improvement strategies.

## Limitations:

Studies reported few details about the intensity, dose, and delivery of QI strategies. Our analysis was limited because the QI strategies were complex and difficult to categorize; some of the strategies overlapped over one another, such as case management and team changes. We did not examine data on patient experience and quality of life, because the focus of our research was the health system. We were unable to summarize information on cost in a meaningful manner, because the information varied widely by context.


## Funding:

This project was funded by the Building Bridges to Integrate Care (BRIDGES) initiative, through the Ontario Ministry of Health and Long-Term Care.


## Summary information:

This summary document was generated by the Knowledge Translation Program in the Li Ka Shing Knowledge Institute at St. Michael's Hospital.

## Effect of quality improvement strategies

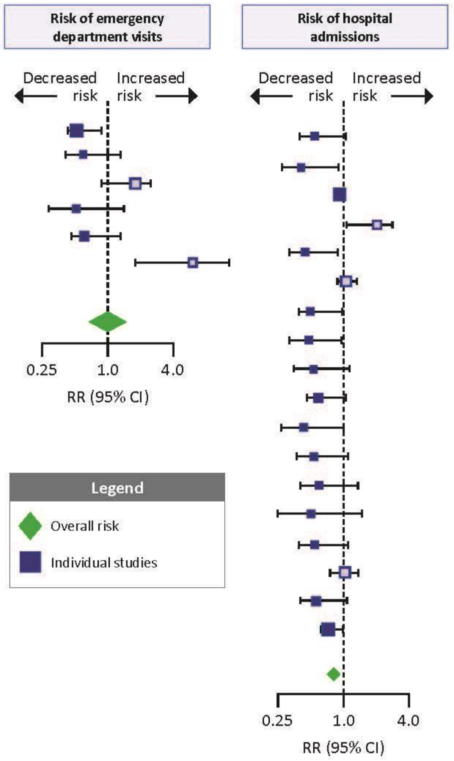


## Definitions:

**RR:** A relative risk of less than 1.0 means that patients involved in a QI strategy had a lower risk of a particular health care event.

**CI:** This [-------] means that we are 95% confident that the true RR lies within the range of values demonstrated by the two brackets.

## Citation:


Tricco, A. C., Antony, J., Ivers, N. M., Ashoor, H. M., Khan, P. A., Blondal, E., Ghassemi, M., MacDonald, H., Chen, M., Ezer, L., Straus, S. E. (2014). Effectiveness of quality improvement strategies for coordination of care to reduce use of health care services: A systematic review and meta-analysis. Canadian Medical Association Journal, 186(15). doi:10.1503/cmaj.140289


---

## Recommended reading list:

Click here for the full list of studies included in this systematic review

**Policy maker novel format (Format B)**

# Quality improvement (QI) strategies reduced hospital admissions among adults with chronic conditions and reduced emergency department (ED) visits among the elderly.

Tricco A, Antony J, Ivers N, Ashoor H, Khan P, Blondal E, Ghassemi M, MacDonald H, Chen M, Ezer L, Straus S.

2014

## Background:

Frequent users of health services are a small group of patients who account for a large amount of hospital admissions and ED visits. There is a need to understand the impact of QI care coordination strategies aimed at reducing the use of these services in this group.

Click here for more information of care coordination strategies

## Key messages for policy and practice:

The key results from this systematic review are similar to previous systematic reviews:

- Patient education and promotion of self-management are effective for improving care
    - These may also be less resource intensive than case management
- Case management and multidisciplinary teams are effective for reducing ED visits
    - These may not be effective for the elderly

**Potential applications:**

- Strategies such as team changes, case management and promotion of self-management may be useful interventions to reduce hospital admissions and ED visits from chronic disease patients and elderly patients.

**Additional interpretations:**

- QI strategies targeting patients (as opposed to clinicians) might be a more efficient use of resources
- Studies on patients with mental illness included comparison groups who were involved in similar QI strategies; it is unclear if these strategies would be effective in patients with mental illness.
- It is unclear how decision makers can best use QI strategies for other patient subgroups and across various settings.

## The most effective QI strategies were:

- **Case management:**
  Diagnosis, treatment and ongoing patient management (e.g., arranging referrals, follow-up of test results, education, reminders) by an individual other than the primary care clinician.
- **Primary care team changes:**
  Changes to the team and how it functions, including routine patient visits with personnel other than the primary care physician, use of multidisciplinary staff and the expansion or revision of team members' professional roles
- **Promotion of self-management:**
  Provision of equipment (e.g., home glucometers) or access to resources (e.g., electronic systems for transferring glucose measurements) and goal-setting to empower patients to manage their own health.
- **Patient education:**
  Educating patients about their disease, including prevention and treatment.

## QI strategies that were not significantly effective:

- Decision support:
  Provision of feedback to clinicians including supportive evidence or organizational supports to facilitate care coordination.

- Clinical information system:
  Distinguished from administrative information systems by the requirement for data entry or data retrieval by clinicians at the point of care.

QI strategies with a patient navigator component (e.g., a staff person who connects patient with doctors and available therapies) were not found to be effective.

## Results:

Elderly patients who were involved in QI strategies (for a median duration of 9 months) were less likely to visit the ED than elderly patients who were not involved in QI strategies.

Fewer patients with chronic conditions who were involved QI strategies (for a median duration of one year) were less likely to be admitted to hospital than patients with chronic conditions who were not involved in QI strategies.

No change in hospital admissions was found among patients with mental illness (e.g., schizophrenia and severe bipolar disorder) who were involved in QI strategies.

## The most effective QI strategies had the following:

- An outreach component that included patient assessment, education, and follow-up conducted outside the clinic or hospital
- A target population that included patients who were the most frequent users of the health care system and those who were at risk of becoming the most frequent users

**Table 1.** The effect of quality improvement strategies on emergency department visits, hospital admissions, clinic visits, and length of stay

| Health care event | Relative Risk (95% Confidence Interval) | Number of included studies |
|---|---|---|
| Emergency department visits | 1.11 (0.65 to 1.90) | 6 |
| Hospital admissions | 0.81 (0.72 to 0.91) | 18 |
| Clinic visits | 0.86 (0.58 to 1.27) | 5 |

*A relative risk of less than 1.0 means that patients involved in a QI strategy had a lower risk of a particular health care event.*

Click here to see the results from different jurisdictions.

## Methods:

We searched MEDLINE, Embase and the Cochrane Library and found 36 randomised control trials (RCTs) and 14 companion reports (total 7494 patients) assessing QI strategies for the coordination of care. We used the Cochrane Effective Practice and Organisation of Care Risk-of-Bias Tool. A meta-analysis was conducted on data reported from RCTs. The included studies were published between 1987 and 2014 in North America, Europe, Australia, Israel, and South Africa.

## Limitations:

Studies reported few details about the intensity, dose, and delivery of QI strategies.

## Funding:

This project was funded by the Building Bridges to Integrate Care (BRIDGES) initiative, through the Ontario Ministry of Health and Long-Term Care.

## Citation:

Tricco, A. C., Antony, J., Ivers, N. M., Ashoor, H. M., Khan, P. A., Blondal, E., Ghassemi, M., MacDonald, H., Chen, M., Ezer, L., Straus, S. E. (2014). Effectiveness of quality improvement strategies for coordination of care to reduce use of health care services: A systematic review and meta-analysis. Canadian Medical Association Journal, 186(15). doi:10.1503/cmaj.140289

## Recommended reading list:

Click here for the full list of studies included in this systematic review

This summary document was generated by the Knowledge Translation Program in the Li Ka Shing Knowledge Institute at St. Michael's Hospital.
