# Supplementary material for: Enhancing the uptake of systematic reviews of effects: what is the best format for health care managers and policy-makers? A mixed-methods study
Source: Implement Sci. 2018 Jun 22;13:84. doi: 10.1186/s13012-018-0779-9 (PMC6014014; doi:10.1186/s13012-018-0779-9)
Supplement: Supplementary file 2 — Figure SA1: Phase 1. Content components that were considered of interest; Figure SA2: Phase 1. Content component modifications that were considered important; Figure SA3: Phase 1. Format features that were considered satisfactory; Figure SA4: Phase 1. Format feature modifications that were considered important; Table SA1: Phase 1. Preference towards font and color; Table SA2: Phase 2. Preference towards color, content, and layout; and Table SA3: Phase 2. Voting results towards color, content, and layout. (DOCX 81 kb) [file 13012_2018_779_MOESM2_ESM.docx]

**Additional file 2 (Figure SA1 to SA4 and Table SA1 to SA3):**

**Figure SA1: Phase 1. Content components that were considered of interest**

**Figure SA2: Phase 1. Content component modifications that were considered important**

**Figure SA3: Phase 1. Format features that were considered satisfactory**

**Figure SA4: Phase 1. Format feature modifications that were considered important**

**Table SA1: Phase 1. Preference towards font and colour**

| **Font & Colour** | ***n (%)*** | |
| --- | --- | --- |
| **Font** |  |  |
| Calibri | 52.0 | 29.1% |
| Arial | 39.0 | 21.8% |
| Times New Roman | 31.0 | 17.3% |
| No preference | 31.0 | 17.3% |
| Cambria | 8.0 | 4.5% |
| Helvetica | 7.0 | 3.9% |
| Unsure | 6.0 | 3.4% |
| Garamond | 5.0 | 2.8% |
|  |  |  |
| **Font size** |  |  |
| Size 12 | 77 | 43.0% |
| Size 11 | 72 | 40.2% |
| No preference | 15 | 8.4% |
| Size 10 | 13 | 7.3% |
| Unsure | 2 | 1.1% |
|  |  |  |
| **Colour** |  |  |
| Blue | 42.0 | 24.1% |
| Unsure | 39.0 | 22.4% |
| Black and white | 34.0 | 19.5% |
| All of the above | 29.0 | 16.7% |
| Green | 8.0 | 4.6% |
| Purple | 8.0 | 4.6% |
| Orange | 7.0 | 4.0% |
| Red | 6.0 | 3.4% |
| Yellow | 1.0 | 0.6% |

**Table SA2: Phase 2. Preference towards colour, content, and layout**

|  | ***n* (%)** | | | | | | | | | | | |
| --- | --- | --- | --- | --- | --- | --- | --- | --- | --- | --- | --- | --- |
| **Systematic review component** | **Total Chosen*** | **Colour** | | | **Layout** | | **Details** | | **Columns** | | **Icon** | |
|  |  | **Grey** | **Blue** | **Green** | **Page 1** | **Page 2** | **More** | **Less** | **1** | **2** | **Yes** | **No** |
| Title | 13 (100.0) | 4 (30.8) | 5 (38.4) | 4 (30.8) | 13 (100.0) | 0 (0.0) | 6 (46.2) | 7 (53.8) | 13 (100.0) | N/A** | 8 (62) | 5 (38) |
| Background: textual blurb | 13 (100.0) | 6 (46.2) | 4 (30.8) | 3 (23.1) | 13 (100.0) | 0 (0.0) | 5 (38.5) | 8 (61.5) | 5 (38.5) | 8 (61.5) | 4 (31) | 9 (69) |
| Background definition of  care coordination | 8 (61.5) | 5 (62.5) | 1 (12.5) | 2 (25.0) | 6 (75.0) | 2 (25.0) | 2 (25.0) | 6 (75.0) | 2 (25.0) | 6 (75.0) | 3 (37) | 5 (63) |
| Recommended reading list | 7 (53.8) | 5 (71.4) | 1 (14.3) | 1 (14.3) | 1 (14.3) | 6 (85.7) | N/A** | N/A** | 7 (100) | N/A** | 2 (29) | 5 (71) |
| Methods: study flow diagram | 12 (92.3) | 4 (33.3) | 6 (50.0) | 2 (16.7) | 8 (66.7) | 4 (33.3) | 5 (41.7) | 7 (58.3) | N/A** | 12 (100.0) | 5 (42) | 7 (58) |
| Results: textual blurb | 8 (61.5) | 5 (62.5) | 3 (37.5) | 0 (0.0) | 6 (75.0) | 2 (25.0) | N/A** | N/A** | N/A** | 8 (100.0) | 8 (100) | N/A** |
| Result: forest plot | 6 (46.2) | 3 (25.0) | 6 (50.0) | 3 (25.0) | 11 (91.7) | 1 (8.3) | N/A** | N/A** | N/A** | 12 (100.0) | 3 (25) | 9 (75) |
| Result: quantitative table | 5 (38.5) | 1 (16.7) | 3 (50.0) | 2 (33.3) | 4 (66.7) | 2 (33.3) | N/A** | N/A** | N/A** | 6 (100.0) | 6 (100) | N/A** |
| Results: effective QI strategies | 9 (69.2) | 1 (20.0) | 3 (60.0) | 1 (20.0) | 2 (40.0) | 3 (60) | N/A** | N/A** | 5 (100.0) | N/A** | 5 (100) | N/A** |
| Results: components of effective QI strategies | 7 (53.8) | 5 (55.6) | 2 (22.2) | 2 (22.2) | 5 (55.6) | 4 (44.4) | N/A** | N/A** | 3 (33.3) | 6 (66.7) | 2 (22) | 7 (78) |
| Results: elements not included in effective QI strategies | 9 (69.2) | 1 (14.3) | 5 (71.4) | 1 (14.3) | 3 (42.8) | 4 (57.1) | 3 (42.9) | 4 (57.1) | 4 (57.1) | 3 (42.9) | 1 (14) | 6 (86) |
| Key Messages | 13 (100.0) | 2 (22.2) | 3 (33.3) | 4 (44.4) | 2 (22.2) | 7 (77.8) | N/A** | N/A** | 9 (100.0) | N/A** | 3 (33) | 6 (67) |
| Limitations | 12 (92.3) | 3 (23.1) | 6 (46.2) | 4 (30.8) | 9 (69.2) | 4 (30.8) | 6 (46.2) | 7 (53.8) | 6 (46.2) | 7 (53.8) | 3 (23) | 10 (77) |
| Funding | 13 (100.0) | 4 (33.3) | 5 (41.6) | 3 (25.0) | 4 (33.3) | 8 (66.7) | 6 (50.0) | 6 (50.0) | 6 (50.0) | 6 (50.0) | 4 (33) | 8 (67) |
| Authors | 10 (76.9) | 7 (53.8) | 3 (23.1) | 3 (23.1) | 3 (23.1) | 10 (76.9) | N/A** | N/A** | 5 (38.5) | 8 (61.5) | 8 (62) | 5 (38) |
| Citations | 12 (92.3) | 7 (70.0) | 2 (20.0) | 1 (10.0) | 7 (70.0) | 3 (30.0) | N/A** | N/A** | 6 (60.0) | 4 (40.0) | 6 (60) | 4 (40) |

*This indicates the number of times participants chose to include the option in their customized systematic review format

** This indicates that there was no option selection for that particular systematic review component

**Table SA3: Phase 2. Voting results towards colour, content, and layout**

|  | ***n* (%)** | | | | | |
| --- | --- | --- | --- | --- | --- | --- |
|  | **Health care managers** | | | **Policy makers/advisors/analysts** | | |
|  | **Option 1** | **Option 2** | **Option 3** | **Option 1** | **Option 2** | **Option 3** |
| Colour | 1 (11.1) | 6 (66.7) | 2 (22.2) | 0 (0.0) | 9 (75.0) | 3 (25.0) |
| Content | 6 (66.7) | 1 (11.1) | 2 (22.2) | 3 (25.0) | 5 (41.7) | 4 (33.3) |
| Layout | 4 (44.4) | 4 (44.4) | 1 (11.1) | 3 (25.0) | 5 (41.7) | 4 (33.3) |
| Overall | 5 (55.6) | 2 (22.2) | 2 (22.2) | 3 (25.0) | 6 (50.0) | 3 (25.0) |
